# Measuring equitable care in multi-hospital markets: A Proportional Share Index Application in New York City

**DOI:** 10.1093/haschl/qxaf088

**Published:** 2025-05-21

**Authors:** Duncan Maru, Deirdre Flynn, Laila Alsabahi, Ana Gallego, Emma Clippinger, Rebecca Friedman, Yogeeta Kuldip, Gavin Myers, Ese Oghenejobo, Amy Shah, Tsu-Yu Tsao, Ewa Wojas, Brian Yim, Michelle Morse

**Affiliations:** Bureau of Equitable Health Systems, New York City Department of Health, New York 11101, United States; Healthcare Payment Financing & Innovation, New York City Department of Health, New York 11101, United States; Healthcare Research and Analytics, New York City Department of Health, New York 11101, United States; Office of Healthcare Planning & Accountability (former), New York City Department of Health, New York 11101, United States; Healthcare System Policy & Legal Strategy, New York City Department of Health, New York 11101, United States; Healthcare System Policy & Legal Strategy, New York City Department of Health, New York 11101, United States; Strategic Planning & Partnerships, New York City Department of Health, New York 11101, United States; Healthcare Justice, New York City Department of Health, New York 11101, United States; Office of Healthcare System Strategy & Accountability, New York City Department of Health, New York 11101, United States; Strategic Planning & Partnerships, New York City Department of Health, New York 11101, United States; Healthcare Research and Analytics, New York City Department of Health, New York 11101, United States; Healthcare Research and Analytics, New York City Department of Health, New York 11101, United States; Healthcare Research and Analytics, New York City Department of Health, New York 11101, United States; Office of the Commissioner of Health, New York City Department of Health, New York 11101, United States

**Keywords:** health equity, hospital business practices, hospital accountability, payer mix, equitable care, proportional share index, PSI, New York City, community benefits, certificate of need, Medicaid, hospital capacity, public health

## Abstract

Community members, elected officials, and policy makers are increasingly calling attention to the issue of inequities in hospital utilization and resource allocation within consolidated multi-hospital markets in the United States. Innovative policy solutions are required to re-shape the incentives driving hospital business practices and behaviors that produce inequitable outcomes and to ensure that equity, as well as economics, drives these business decisions. New measures can drive evidence-informed policy making and track the impact of new laws, regulations, and practices. In this paper, we illustrate the development and potential applications of the Proportional Share Index (PSI) using New York City (NYC) as a case study, highlighting its ability to quantify and track equitable access to hospitals across multi-hospital markets. The PSI incorporates both a measure of those who are covered by Medicaid or who are self-pay (largely uninsured) and hospital capacity in determining whether a given hospital is providing its proportionate, fair amount of care to these populations. We discuss how the PSI and related measures may inform policy interventions aimed at mitigating health inequities.

## Health equity and hospitals

Hospital admission decisions—who gets admitted, where, for what conditions, and for how long—are shaped by the policies, regulations, and financial incentives that drive hospital business practices and behaviors, often leading to an inequitable distribution of resources.^1^ Ensuring equitable allocation of inpatient bed capacity is crucial, especially for individuals covered by Medicaid or those who are uninsured. Here, we develop an approach to quantify a hospital's contribution to addressing the hospitalization needs of its community. We aim to better understand how equitably existing resources are distributed given their critical implications for population health.^2^

In a previous article, we described how unequal distribution of hospital resources during the first wave of COVID-19 contributed to differences in patient outcomes.^3^ This issue is not just related to pandemics, with research demonstrating that it characterizes hospital care more generally.^4-6^ Here, we examine the non-profit acute care hospitals in NYC, where demonstrated disparities in care highlight the urgent need for better measures to understand and address this issue.

In New York State, all acute care hospitals are non-profit by law.^7^ This means that while chronically resource-depleted safety net hospitals, which provide essential care to those covered by Medicaid or who are uninsured,^8^ receive public subsidies, so do hospitals that are rich in financial and physical assets (such as advanced equipment or impressive public spaces). The unequal distribution of resources is compounded by the ability of large healthcare systems to choose which, if any, Medicaid health plans to accept, which outpatient networks feed their inpatient care, and how to staff clinics (often a source of inpatient referrals) to differentiate wait times depending on insurance status. These inequities have led to active conversations and research around increasing hospital accountability to equitably serving patients in their region regardless of insurance status.^9^

One measure of a hospital's business practices related to serving patients is payer mix. Payer mix has many different definitions. It can describe the proportion of any of the following: “revenues, charges, discharges or patient days from different medical insurance” payers, like Medicare, Medicaid, or commercial plans.^10^ Given the varying profitability of these different payer types, these metrics are widely used internally for business decisions and financial planning by hospitals. However, as KFF notes, “we are unaware of any public data with comprehensive and consistently defined information on payer mix…that covers all hospitals and health systems in the country.”^11^ Since consistent definitions and comprehensive data sources for payer mix do not exist, its application by public health authorities or researchers attempting to understand health inequities has been limited.

Another measure of hospital commitment to equitable care is the Hospitals Index created by the Lown Institute. Using nonprofit hospital IRS 990 tax filings, the Lown Institute calculated a metric called “fair share spending,” which compares the estimated value of hospital tax exemptions to the amount spent on what Lown considers “meaningful” community investments.^12^ The strength of this measure for policy and narrative change is that it provides a clear and simple financial accounting of how much hospitals “give” and “take.” This provides salience for elected officials and regulators who are trying to understand the return on investment of the substantial public subsidies provided by a hospital's nonprofit status.

Medicaid Disproportionate Share (DSH) payments are a long-standing federal policy tool designed to “improve access for Medicaid and uninsured patients as well as the financial stability of safety-net hospitals.”^13^ In theory, the amount of money a hospital receives in DSH payments could serve as a useful metric for assessing its commitment to equitable care. However, these payments have faced significant criticism, with concerns that the methodology has “been warped to benefit other (often better-resourced) providers” over time.^14^ Additionally, inconsistencies in how these payments are calculated across states further limit their usefulness in evaluating the equitable allocation of hospital resources.

Complementing these measures, we describe here the Proportional Share Index (PSI). We intend for the PSI to be a straightforward measure of hospital accountability to providing a proportionate share of care to those covered by government programs such as Medicaid or who are self-pay (largely uninsured). We apply the PSI to the NYC hospital ecosystem to demonstrate its relationship to issues of health equity and to identify performance variations within the existing healthcare system. We describe examples of how our local health department can utilize the PSI to monitor, mitigate, and ultimately prevent the public health harms related to inequitable access to care based on insurance type.

## Introducing the Proportional Share Index (PSI)

With the Proportional Share Index (PSI), we aim to quantify a hospital's contribution to serving the market's hospitalization needs. The PSI is a measure that illustrates the distribution of Medicaid and self-pay discharges relative to the distribution of bed capacity among hospitals in the service area of interest.


$$\;Capacity\;share\;for\;hospital\;i\;=\frac{Number\;of\;licensed\;beds\;for\;hospital\;i\;}{Total\;number\;of\;licensed\;beds\;in\;service\;area}$$

$$\;Medicaid\;or\;self\text{-}pay\;for\;hospital\;i\;=\frac{Number\;of\;Medicaid\;or\;self\text{-}pay\;discharges\;for\;hospital\;i}{Total\;number\;of\;Medicaid\;or\;self\text{-}pay\;discharges\;in\;service\;area}$$

$$\;\begin{array}{rl} PSI & \;for\;Hospital\;i\\ & =\;\frac{Medicaid\;and\;self\text{-}pay\;share\;for\;hospital\;i}{Capacity\;share\;for\;hospital\;i} \end{array}$$

By definition, NYC as a healthcare market has a PSI of 1. Thus, any hospital with a PSI of less than 1 may be interpreted as underserving individuals who are covered by Medicaid or who are uninsured. To analyze the PSI for NYC, we use publicly available 2022 data from the New York Statewide Planning and Research Cooperative System (SPARCS), which is a comprehensive all-payer data reporting system that collects discharge data from all hospitals. For PSI, we examined the total inpatient discharges and identified how many of those discharges were attributed to Medicaid and self-pay for all hospitals in NYC. In addition to SPARCS data, we used facility certified bed information for NYC hospitals to create a measure of hospital capacity. Although data on certified beds is publicly available and updated daily, we used an internal 2022 bed file provided by New York State Department of Health to ensure consistency with the 2022 SPARCS data and mitigate discrepancies due to changes in certified bed counts. The critical difference between PSI and looking at discharge percentages by payer alone is that PSI incorporates the number of certified beds, a fixed asset, as the measure of capacity. It avoids penalizing hospitals that attract disproportionately high volumes of commercial patients if their share of service to the Medicaid and uninsured population aligns with their share of licensed bed capacity. Additionally, we chose to evaluate certified beds over length of stay, as our primary concern in this paper is with the issue of equitable access to hospital care rather than duration of treatment.

## Applying PSI to the New York City hospital market

The NYC hospital ecosystem is diverse and expansive, encompassing a range of public, private, academic, and specialized medical centers. These hospitals serve a vast and varied population, playing a crucial role in the city's healthcare infrastructure. Within this ecosystem, hospitals differ in their approaches to promoting health equity, with some focusing on culturally sensitive community outreach programs and partnerships to address inequities in access and care, while others prioritize research.

**Figure 1.**
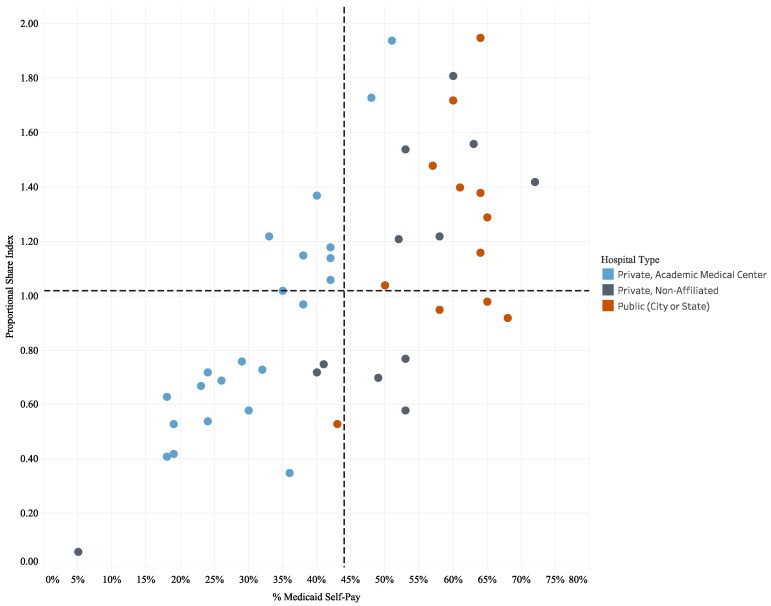
Hospital Proportional Share Index (PSI) by Hospital Type in NYC, 2022. Source: New York State Department of Health, Health Facility 2022 Certification Information, & Statewide Planning and Research Cooperative System (SPARCS), Hospital Inpatient Discharges (SPARCS De-Identified), 2022, September 2024 release. Notes: Children’s hospitals, specialty hospitals, hospitals affected by closure (eg, Kingsbrook), and hospitals without intensive care units were excluded from the analysis (*n* = 12).

Figure 1 illustrates the Proportional Share Index for 46 hospitals by hospital type in NYC in 2022. A hospital is categorized as either a Private Academic Medical Center (AMC) or an affiliate of an AMC, a Private hospital unaffiliated with an AMC, or a Public hospital. The scatter plot is divided into four quadrants, which reflect the PSI and the percentage of Medicaid and self-pay discharges. The dashed line indicates the citywide average of the percentage of Medicaid and self-pay discharges of 44%. The Appendix details hospital-level data including facility certified bed information, inpatient volume, PSI, payer mix, and race/ethnicity.

We chose to analyze individual hospitals rather than hospital systems to provide greater transparency around an individual hospital's share of service delivery. As the graph demonstrates, many of NYC's resource-rich private AMCs and their affiliates have a PSI of less than one, indicating they do not serve a proportionate share of the city's Medicaid and self-pay patients relative to their capacity. Montefiore is a notable exception as the only private AMC where all hospitals in its system have a PSI greater than 1. In contrast, most of the hospitals that are part of the public system have a PSI of 1 or greater, indicating that public hospitals are a reliable health system for Medicaid and self-pay patients.

Table 1 illustrates the average PSI by hospital type for all NYC hospitals included in our analysis. Of the 46 NYC hospitals included in our analysis, just under half are private AMCs or their affiliates (*n* = 22), with 55% of the licensed bed share in the city. However, on average, these hospitals only serve 48% of the total Medicaid or self-pay population, resulting in a PSI of.87. In contrast, the 12 Public institutions have the highest average PSI of 1.21, which is expected given their disproportionate service to Medicaid and self-pay patients (28%) vs their licensed bed capacity (23%).

**Table 1.** PSI calculations by Hospital Type in NYC, 2022.

Hospital inpatient discharges (Public SPARCS 2022)	Academic medical center and affiliates	Private, non-affiliated	Public city/state	NYC total
Number of hospitals	22	12	12	46
Total inpatient discharges(a)	549 735	163 632	171 325	884 692
% Black or Hispanic	42%	57%	65%	49%
Medicaid and self-pay	176 460 (b)	88 306 (b)	103 701 (b)	368 467 (c)
% Medicaid and self-pay (b ÷ a)	32%	54%	61%	42%
Certified beds	12 023 (d)	4693 (d)	5065 (d)	21 781 (e)
Medicaid and self-pay share (b ÷ c)	48%	24%	28%	
Certified beds share (d ÷ e)	55%	22%	23%	
PSI (Medicaid and self-pay share/certified beds share)	0.87	1.11	1.21	

Source: New York State Department of Health, Health Facility 2022 Certification Information, & Statewide Planning and Research Cooperative System (SPARCS), Hospital Inpatient Discharges (SPARCS De-Identified), 2022, September 2024 release. Notes: AMC (Academic Medical Center). Children’s hospitals, specialty hospitals, hospitals affected by closure (eg, Kingsbrook), and hospitals without intensive care units were excluded from the analysis (*n* = 12).

## PSI and racial and ethnic health inequities

We developed the PSI to support our commitment to creating actionable policy levers that can address inequities in hospitalization and outcomes by race, ethnicity, and insurance status. While the PSI does not directly measure race, the strong correlation between race and health insurance status suggests that findings of disparate access based on insurance status may also indicate inequitable access to care across racial groups.^15^

**Figure 2.**
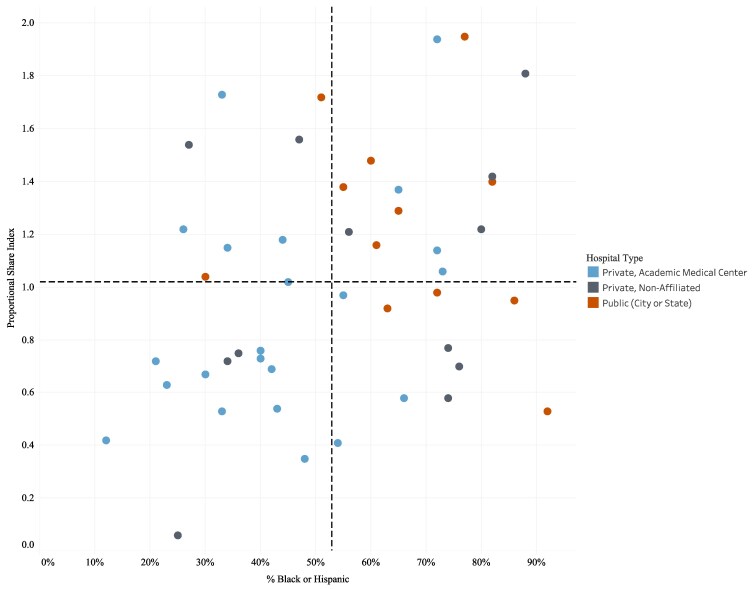
Hospital Proportional Share Index (PSI) by race/ethnicity and Hospital Type in NYC, 2022. Source: New York State Department of Health, Health Facility 2022 Certification Information & Statewide Planning and Research Cooperative System (SPARCS), Hospital Inpatient Discharges (SPARCS De-Identified), 2022; September 2024 release. Notes: Children’s hospitals, specialty hospitals, hospitals affected by closure (eg, Kingsbrook), and hospitals without intensive care units were excluded from the analysis (*n* = 12).

In NYC, Medicaid beneficiaries are disproportionately people of color. As shown in Figure 2, Black and Hispanic residents, as well as those who are covered by Medicaid or are self-pay, comprise most of the discharges of the city's public institutions. In contrast, White and commercially insured residents tend to disproportionately use the city's private AMCs and their affiliates. This conforms to earlier research, which demonstrated that access to AMCs was limited for marginalized populations.^16^

Medicare Cost Report data made available through The National Academy for State Health Policy (NASHP) Hospital Cost Tool also illustrates concerning disparities.^17^ Using the Hospital Cost Tool, we found that the 7 hospitals with the largest operating margins per adjusted patient discharge in 2021—all AMCs or their affiliates—accounted for 46% of the City's bed capacity but only served 33% of Medicaid discharges. Other research has shown that hospitals serving people of color have fewer capital assets than other hospitals.^18^ An analysis of the PSI could be used alongside analyses like these in the effort to better understand a hospital's commitment to equitable care and to encourage the changes in business practice that would be necessary to rectify the inequities.

Figure 2 compares the Proportional Share Index of NYC hospitals relative to the percentage of Black or Hispanic individuals served. The dashed line indicates the citywide average percentage of Black and Hispanic patients served of 54%.

Although the PSI does not measure race, we observe striking racial inequities. Ten of the 13 hospitals in the lower left quadrant are AMCs or their affiliates. These hospitals serve less than a proportionate share of Medicaid and self-pay patients based on bed capacity and serve less than the citywide average of Black and Hispanic residents. The public hospitals are concentrated in the upper right quadrant, showing their disproportionately high share of service to both Medicaid and self-pay patients, as well as to NYC's Black and Hispanic residents.

**Figure 3.**
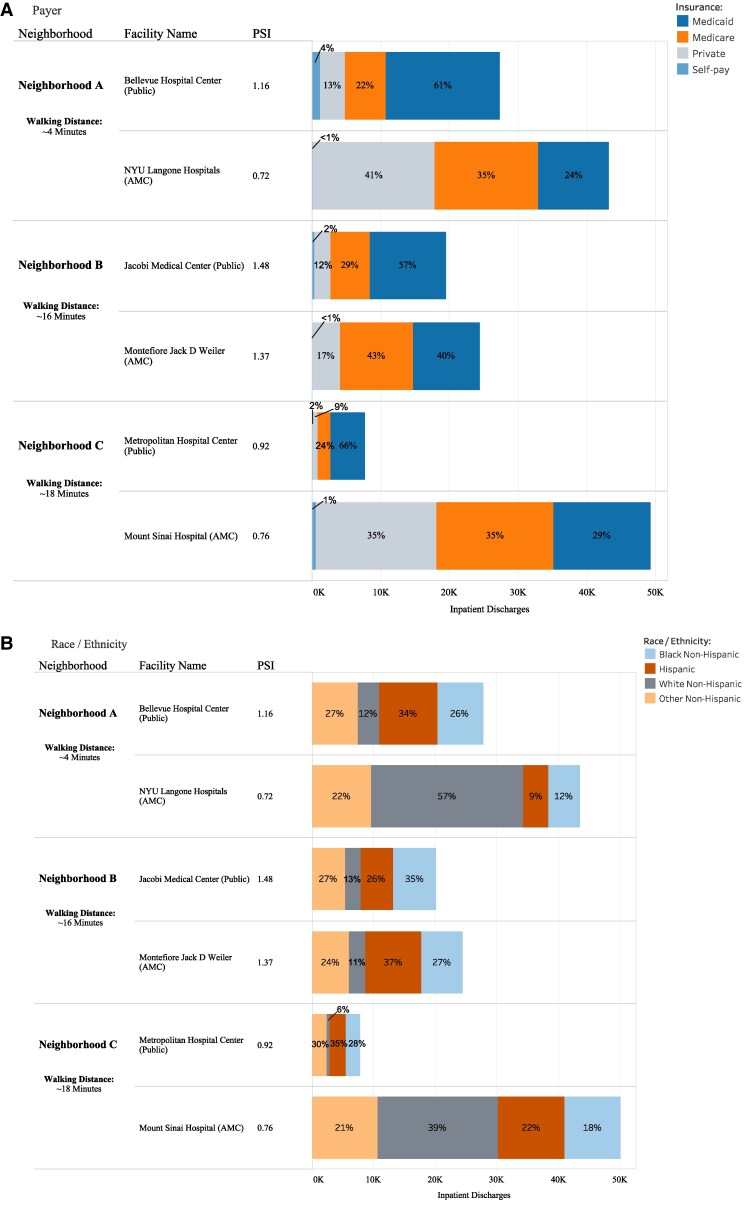
Inpatient discharges by Hospital Type in NYC, 2022: Payer (A), Race/Ethnicity (B). Source: New York State Department of Health, Health Facility 2022 Certification Information & Statewide Planning and Research Cooperative System (SPARCS), Hospital Inpatient Discharges (SPARCS De-Identified), 2022; September 2024 release. Notes: Asian/Pacific Islander race was unavailable. “Other” payer was excluded. AMC (Academic Medical Center).

Figure 3 demonstrates that geography alone cannot explain differences in the PSI between public hospitals and AMCs. By comparing three pairs of neighboring hospitals within a 4- to 18-minute walk of each other, we observe differences in inpatient discharges by payer (Figure 3/Panel A) and race/ethnicity (Figure 3/Panel B) between the public hospital and the nearby AMC.

In Neighborhood A in Manhattan, Bellevue (Public) had a PSI of 1.16, with 61% of discharges for Black or Hispanic patients and 65% Medicaid or self-pay, compared to NYU (AMC), which had a PSI of 0.72, with 21% of discharges for Black or Hispanic patients and 24% Medicaid or self-pay. In Neighborhood B in the Bronx, both hospitals had a PSI greater than 1 and similar proportions of discharges for Black or Hispanic patients, but Jacobi (Public) had a higher share of Medicaid or self-pay discharges at 59%, compared to 40% at Montefiore Jack D Weiler (AMC). In Neighborhood C, also in Manhattan, both hospitals had a PSI below 1, yet Metropolitan (Public) had 63% of discharges for Black or Hispanic patients and 68% Medicaid or self-pay, compared to Mount Sinai (AMC) at 30% and 40%, respectively.

## Monitor, mitigate, prevent: local health department action to increase hospital options for all New Yorkers

There are many regulatory tools and approaches that can be used to influence health equity. Catalyst for Payment Reform groups these activities into four categories: banning/punishing bad behavior, preventing further erosion of competition, regulating costs/prices, and building infrastructure.^19^ Despite bearing substantial fiscal and health burdens of inequitable health systems that serve over 40% of New York State's population, New York City’s local government has minimal regulatory or payment authority. Measures that contribute to our understanding of collective accountability to health equity and facilitate simple comparisons between hospitals, such as the PSI, can help close these gaps in public oversight and improve accountability of hospitals, the boards that govern them, and the executives that manage them.

Our goal is for all New York City hospitals to achieve a PSI of 1. By setting this goal, we aim to encourage hospitals with PSIs <1 to build stronger pipelines for hospital utilization in neighborhoods populated by individuals disproportionately insured by Medicaid. This could contribute to Medicaid populations experiencing decreased wait times for services and greater integration at all types of NYC hospitals. There are many opportunities for these hospitals to expand their reach, either directly or through partnerships with trusted providers in marginalized communities, which would both increase the PSI and increase the equitable distribution of hospital resources.

In this section, we discuss how the PSI can be further applied to our efforts, and those of other local health departments, to monitor, mitigate, and prevent healthcare inequities in the absence of greater regulatory authority.

## Monitoring: longitudinal tracking

Increased interest from federal and state regulators and employers in hospital and insurer price transparency, as well as hospital system financial health, has spurred the development of publicly accessible data sources and tools, such as the Employers Forum of Indiana's Sage Transparency website and NASHP's Hospital Cost Tool.^17,20^ Locally, New York City Council passed legislation requiring transparency in hospital business practices that contribute to healthcare costs, which can create barriers to equity and access, and the NYC Department of Health released its inaugural annual report related to this topic.^21^ These efforts to increase hospital accountability have the potential to significantly influence the hospital industry. Local health departments can use these new tools and data sources, including the PSI, to track changes in the distribution of Medicaid discharges across local hospitals over time, and more broadly, to understand a hospital's commitment to providing equitable care. Combined with longitudinal monitoring of other environmental factors that can influence the PSI, such as changes in demographics or the competitive landscape, these analyses can provide critical insights into how these dynamics affect equitable access to care based on insurance type.

## Mitigating: community benefits

To maintain their tax-exempt status and receive taxpayer funded incentives such as tax exemptions, graduate medical education incentives, and DSH payments, nonprofit hospitals are required to provide a benefit to the community they serve.^12,22-24^ These Community Benefit requirements are one of the few mechanisms available to mitigate the negative health equity impacts resulting from surplus-maximizing decisions by healthcare institutions. However, these requirements have not effectively incentivized nonprofit hospitals to adequately serve Medicaid patients or those who are uninsured, contributing to the inequitable results highlighted by our PSI analysis.^25^

There is ongoing discussion about improving the reporting of Community Benefits, including establishing clearer standards for charity care eligibility, refining the definition of what constitutes a Community Benefit, and requiring greater hospital participation in public health programs.^26^ The effort to improve Community Benefit reporting could be enhanced by incorporating a metric like the PSI, which offers a straightforward means to measure non-safety net hospitals' relative provision of services to the Medicaid and uninsured population. While improvements in Community Benefit reporting are being debated, local health departments could use the PSI immediately to better understand the commitment to and encourage the provision of community-benefitting health services by hospitals to equitably serve Medicaid and uninsured patients according to their capacity.^27^

## Preventing: Certificate of Need applications

The PSI can also serve as a measure of accountability within the Certificate of Need (CON) process, which requires certain health care facilities, including hospitals, to demonstrate the public need of proposed projects such as construction of new facilities before receiving state approval. As of June 2023, certain healthcare facility projects in New York State must include a Health Equity Impact Assessment (HEIA) as part of the CON process.^28^ The HEIA process aims to evaluate how a proposed project could impact health equity and medically underserved groups. As CON applications often include requests for changes in bed composition, PSI might be particularly relevant to that evaluation process, especially as we use licensed beds, which represents maximum capacity, for the analysis. By providing the PSI for the hospital in question, the NYC Health Department can offer a clear and standardized metric that demonstrates how a proposed project might affect health equity, thus providing data-driven support to prevent projects that could lead to less equitable access to care. For example, if the PSI of a given hospital is <1, the hospital may need to more closely consider how or if the closure of inpatient beds might impact its PSI and should describe how it intends to address this issue in its HEIA mitigation plan. Conversely, for a hospital with a PSI >1, any proposed expansion of beds would likely be looked upon favorably, by creating opportunities to serve more individuals on Medicaid or without insurance.

## Limitations and future developments

Currently, the PSI has several limitations. It was developed using the NYC hospital ecosystem, potentially limiting its applicability to other hospital markets. It is limited to understanding relative disparities in hospital discharges and not to understanding any unmet hospital needs resulting from systemic barriers to accessing care. Additionally, as hospitals transition to providing more care in outpatient settings, it is unclear how this will affect the PSI, which focuses on *inpatient* beds.^29^ Similar adjustments to the PSI methodology may be required to maintain its relevance. Additionally, PSI uses licensed beds rather than staffed beds. To the extent that a hospital has many beds that are unstaffed, the PSI for that hospital would be understated. Also, hospitals may be able to achieve a PSI greater than 1 by increasing the overall number of patients they see and discharge without changing the payer mix, which could have implications for the quality of care. Similarly, hospitals could decommission beds to influence the calculations. We also recognize that a low PSI could indicate either a lower number of discharges or a higher length of stay (LOS). A higher LOS often correlates with patients who have more complex needs, requiring extended care due to the severity of their conditions or the intensity of services provided. While a low PSI might suggest lower utilization, it could also reflect higher patient complexity. These factors highlight the importance of considering not only volume but also the intensity of care when advocating for equitable access. Finally, the PSI will change based on the service area analyzed because both the total number of discharges and the total number of certified beds will change. While this means that the PSI can be used to analyze, for example, the equitable distribution of hospital resources within a given neighborhood, using an independent metric, like the administrative boundary of a county or borough line, can remove some of the risk of bias in the analysis.

In addition, the PSI should be applied cautiously given the precarious financial situation of many safety net hospitals. These hospitals need sufficient resources to maintain the necessary level of care for their patients. Therefore, the PSI should not be a metric of relevance for hospitals that are financially distressed, or whose Medicaid payer mix is significantly higher than the jurisdiction average. One possible unintended effect of achieving a PSI of 1 across all hospitals is that safety net institutions might see a reduction in the number of patients they serve, as these patients seek other care options. This could further undermine the viability of the safety net if it is not paired with the level of investment that government and private actors owe to these hospitals to compensate for decades of disinvestment. This risk, while real, should be proactively planned for rather than maintaining an inequitable status quo.

Despite these limitations, the PSI is a valuable tool to support monitoring, mitigating, and preventing healthcare system inequities and related harms. The PSI, with its simple calculation and use of publicly available data, can enhance our understanding of a hospital's commitment to equitable care by providing a new interpretation of service delivery. In the future, use cases could be developed for ambulatory care services as well by adapting the capacity denominator to include cancer care, primary care, and behavioral health. We believe the PSI can add an important perspective alongside other approaches to deepen our collective understanding of equitable access to healthcare for all New Yorkers.

## Supplementary Material

qxaf088_Supplementary_Data
