# Supplementary material for: Measuring equitable care in multi-hospital markets: A Proportional Share Index Application in New York City
Source: Health Aff Sch. 2025 May 21;3(5):qxaf088. doi: 10.1093/haschl/qxaf088 (PMC12093469; doi:10.1093/haschl/qxaf088)
Supplement: qxaf088_Supplementary_Data [file qxaf088_supplementary_data.zip › Appendix.pdf]

Appendix: NYC Hospital Bed Capacity, Discharges, Payer Mix, Race/Ethnicity, and Proportional Share Index (PSI) 2022

PFI	Facility Name	Hospital Borough	Hospital type	Total Beds 2022	Total Inpatient Discharges 2022	Proportional Share Index (PSI)	% White	% Black or Hispanic	% Medicaid or Self-pay	% Medicare	% Private
1165	Jacobi Medical Center	Bronx	Public (City or State)	457	20,095	1.48	13%	60%	57%	28%	22%
1168	Montefiore Medical Center-Wakefield Hospital	Bronx	Affiliated w/Private Academic Medical Center	321	14,790	1.14	7%	72%	42%	42%	38%
1169	Montefiore Medical Center - Henry & Lucy Moses	Bronx	Affiliated w/Private Academic Medical Center	816	35,093	1.06	9%	73%	42%	39%	45%
1172	Lincoln Medical & Mental Health Center	Bronx	Public (City or State)	362	18,477	1.95	2%	77%	64%	26%	12%
1176	SBH Health System	Bronx	Private, Non-Affiliated	422	13,998	1.42	15%	82%	72%	21%	9%
1178	BronxCare Hospital Center	Bronx	Private, Non-Affiliated	415	21,106	1.81	2%	88%	60%	33%	11%
1186	North Central Bronx Hospital	Bronx	Public (City or State)	213	7,079	1.29	7%	65%	65%	24%	17%
1286	Brookdale Hospital Medical Center	Brooklyn	Private, Non-Affiliated	530	13,098	0.77	5%	74%	53%	30%	23%
1288	Brooklyn Hospital Center - Downtown Campus	Brooklyn	Private, Non-Affiliated	464	11,381	0.70	8%	76%	49%	34%	36%
1293	New York Community Hospital of Brooklyn, Inc	Brooklyn	Private, Non-Affiliated	134	2,582	0.06	64%	25%	5%	73%	254%
1294	Coney Island Hospital	Brooklyn	Public (City or State)	371	12,943	1.04	44%	30%	50%	40%	20%
1301	Kings County Hospital Center	Brooklyn	Public (City or State)	624	17,446	0.95	4%	86%	58%	27%	26%
1304	NYU Langone Hospital-Brooklyn	Brooklyn	Affiliated w/Private Academic Medical Center	444	27,020	1.73	37%	33%	48%	35%	34%
1305	Maimonides Medical Center	Brooklyn	Private, Non-Affiliated	711	34,581	1.54	49%	27%	53%	34%	24%
1306	New York Presbyterian Brooklyn Methodist Hospital	Brooklyn	Affiliated w/Private Academic Medical Center	591	29,155	1.02	30%	45%	35%	37%	85%
1309	Interfaith Medical Center	Brooklyn	Private, Non-Affiliated	287	5,263	0.58	6%	74%	53%	31%	21%
1318	Wyckoff Heights Medical Center	Brooklyn	Private, Non-Affiliated	324	11,490	1.22	10%	80%	58%	32%	17%
1320	University Hospital of Brooklyn	Brooklyn	Public (City or State)	342	7,183	0.53	3%	92%	43%	40%	37%
1324	Mount Sinai Brooklyn	Brooklyn	Affiliated w/Private Academic Medical Center	212	8,100	0.41	35%	54%	18%	68%	78%
1437	New York Presbyterian Lower Manhattan Hospital	Manhattan	Affiliated w/Private Academic Medical Center	180	8,859	0.67	23%	30%	23%	27%	205%
1438	Bellevue Hospital Center	Manhattan	Public (City or State)	912	27,761	1.16	12%	61%	64%	21%	22%
1439	Mount Sinai Beth Israel	Manhattan	Affiliated w/Private Academic Medical Center	696	11,628	0.35	28%	48%	36%	48%	47%
1445	Harlem Hospital Center	Manhattan	Public (City or State)	268	10,448	1.40	4%	82%	61%	29%	15%
1450	Lenox Hill Hospital	Manhattan	Affiliated w/Private Academic Medical Center	632	30,206	0.53	46%	33%	19%	37%	246%
1454	Metropolitan Hospital Center	Manhattan	Public (City or State)	338	7,760	0.92	6%	63%	68%	24%	13%
1456	Mount Sinai Hospital	Manhattan	Affiliated w/Private Academic Medical Center	1,139	50,110	0.76	39%	40%	29%	34%	124%
1458	New York Presbyterian New York Weill Cornell Center	Manhattan	Affiliated w/Private Academic Medical Center	744	43,877	0.63	48%	23%	18%	28%	307%
1463	NYU Langone Hospitals	Manhattan	Affiliated w/Private Academic Medical Center	844	43,482	0.72	57%	21%	24%	35%	174%
1464	New York Presbyterian Columbia Presbyterian Center	Manhattan	Affiliated w/Private Academic Medical Center	1,022	44,167	0.97	27%	55%	38%	32%	85%
1466	Mount Sinai West	Manhattan	Affiliated w/Private Academic Medical Center	514	22,949	0.69	41%	42%	26%	28%	178%
1469	Mount Sinai Morningside	Manhattan	Affiliated w/Private Academic Medical Center	495	15,975	0.58	18%	66%	30%	52%	59%
1626	Elmhurst Hospital Center	Queens	Public (City or State)	545	19,797	1.38	12%	55%	64%	23%	18%
1628	Flushing Hospital Medical Center	Queens	Private, Non-Affiliated	299	12,416	1.56	14%	47%	63%	25%	18%
1629	Jamaica Hospital Medical Center	Queens	Private, Non-Affiliated	402	15,903	1.21	13%	56%	52%	34%	24%
1630	Long Island Jewish Medical Center	Queens	Affiliated w/Private Academic Medical Center	1,015	39,078	0.73	33%	40%	32%	37%	97%
1633	Queens Hospital Center	Queens	Public (City or State)	269	13,047	1.72	6%	51%	60%	28%	21%
1635	St Johns Episcopal Hospital So Shore	Queens	Private, Non-Affiliated	257	7,823	0.72	20%	34%	40%	48%	17%
1637	New York - Presbyterian/Queens	Queens	Affiliated w/Private Academic Medical Center	535	27,590	1.15	22%	34%	38%	43%	52%
1638	Long Island Jewish Forest Hills	Queens	Affiliated w/Private Academic Medical Center	312	14,966	1.18	27%	44%	42%	40%	44%
1639	Mount Sinai Hospital - Mount Sinai Hospital of Queens	Queens	Affiliated w/Private Academic Medical Center	228	8,809	0.54	33%	43%	24%	61%	66%
1692	Woodhull Medical & Mental Health Center	Brooklyn	Public (City or State)	364	9,289	0.98	9%	72%	65%	26%	13%
1737	Staten Island University Hospital Prince's Bay	Staten Island	Affiliated w/Private Academic Medical Center	194	7,196	0.42	80%	12%	19%	60%	104%
1738	Richmond University Medical Center	Staten Island	Private, Non-Affiliated	448	13,991	0.75	46%	36%	41%	27%	81%
1740	Staten Island University Hosp-North	Staten Island	Affiliated w/Private Academic Medical Center	472	29,607	1.22	64%	26%	33%	46%	57%
3058	Montefiore Med Center - Jack D Weiler	Bronx	Affiliated w/Private Academic Medical Center	421	24,448	1.37	11%	65%	40%	43%	42%
3975	New York Presbyterian- Allen Hospital	Manhattan	Affiliated w/Private Academic Medical Center	196	12,630	1.94	15%	72%	51%	36%	26%

Source: New York State Department of Health, Health Facility 2022 Certification Information & Statewide Planning and Research Cooperative System (SPARCS), Hospital Inpatient Discharges (SPARCS De-Identified), 2022; September 2024 release.
Notes: AMC (Academic Medical Center). Children's hospitals, specialty hospitals, hospitals affected by closure (e.g., Kingsbrook), and hospitals without intensive care units were excluded from the analysis (n=12).
